# Genetic Correction of Tauopathy Phenotypes in Neurons Derived from Human Induced Pluripotent Stem Cells

**DOI:** 10.1016/j.stemcr.2013.08.001

**Published:** 2013-08-29

**Authors:** Helen Fong, Chengzhong Wang, Johanna Knoferle, David Walker, Maureen E. Balestra, Leslie M. Tong, Laura Leung, Karen L. Ring, William W. Seeley, Anna Karydas, Mihir A. Kshirsagar, Adam L. Boxer, Kenneth S. Kosik, Bruce L. Miller, Yadong Huang

**Affiliations:** 1 Gladstone Institute of Neurological Disease, San Francisco, CA 94158, USA; 2 Department of Neurology, University of California, San Francisco, CA 94143, USA; 3 Biomedical Sciences Graduate Program, University of California, San Francisco, CA 94143, USA; 4 Memory and Aging Center, University of California, San Francisco, CA 94143, USA; 5 Department of Pathology, University of California, San Francisco, CA 94143, USA; 6 Neuroscience Research Institute and Department of Molecular, Cellular, and Developmental Biology, University of California, Santa Barbara, CA 93106, USA

## Abstract

Tauopathies represent a group of neurodegenerative disorders characterized by the accumulation of pathological TAU protein in brains. We report a human neuronal model of tauopathy derived from induced pluripotent stem cells (iPSCs) carrying a TAU-A152T mutation. Using zinc-finger nuclease-mediated gene editing, we generated two isogenic iPSC lines: one with the mutation corrected, and another with the homozygous mutation engineered. The A152T mutation increased TAU fragmentation and phosphorylation, leading to neurodegeneration and especially axonal degeneration. These cellular phenotypes were consistent with those observed in a patient with TAU-A152T. Upon mutation correction, normal neuronal and axonal morphologies were restored, accompanied by decreases in TAU fragmentation and phosphorylation, whereas the severity of tauopathy was intensified in neurons with the homozygous mutation. These isogenic TAU-iPSC lines represent a critical advancement toward the accurate modeling and mechanistic study of tauopathies with human neurons and will be invaluable for drug-screening efforts and future cell-based therapies.

## Highlights

• A human neuron model of tauopathy using TAU-A152T-iPSCs
• Correction of TAU-A152T mutation eliminates tauopathy
• Engineered homozygous TAU-A152T mutation intensifies tauopathy
• A152T mutation increases TAU proteolysis, leading to tauopathy

## Introduction

Tauopathies are a group of neurodegenerative disorders characterized by the accumulation and aggregation of the pathological TAU protein in human brains (Hutton, 2000; Lee et al., 2001; Mandelkow and Mandelkow, 2012; Vossel and Miller, 2008). TAU, encoded by the *MAPT* gene on chromosome 17, is a microtubule binding protein that is highly expressed in neurons and predominantly located in neuronal axons (Goedert et al., 1989; Kosik et al., 1989; Morris et al., 2011; Neve et al., 1986). Hyperphosphorylation and aggregation of TAU cause neurofibrillary pathologies, including tangles and neuropil threads that are often seen in brains of patients with Alzheimer’s disease (AD) (Hutton, 2000; Kosik et al., 1986; Lee et al., 2001; Mandelkow and Mandelkow, 2012; Vossel and Miller, 2008). Tauopathies are also typical of other neurodegenerative disorders, including frontotemporal dementia (FTD) and progressive supranuclear palsy (PSP) (Hutton, 2000; Lee et al., 2001; Mandelkow and Mandelkow, 2012; Vossel and Miller, 2008). Most FTD and PSP cases are sporadic, but a minority are familial (Weder et al., 2007). FTD and PSP syndromes can be caused by mutations in the *MAPT* gene that result in abnormal TAU phosphorylation and aggregation, leading to neurodegeneration.

Transgenic murine models of tauopathies have revealed fundamental insights into the disease (Lee et al., 2001; Mandelkow and Mandelkow, 2012), but their value as predictive preclinical models is unknown. In fact, many candidate drugs successful in rodent models of neurodegenerative diseases have failed in humans (Ashe and Zahs, 2010; Huang and Mucke, 2012). Thus, new humanized disease models, such as mutation- and patient-specific induced pluripotent stem cells (iPSCs), are urgently needed for further development of therapeutic strategies for tauopathies.

Human iPSCs are a highly promising approach for investigating cellular properties of traditionally challenging neurodegenerative disorders (HD iPSC Consortium, 2012; Israel et al., 2012; Kondo et al., 2013; Park et al., 2008; Yamanaka, 2009). Because postmortem tissue represents late-stage disease, modeling tauopathy onset and progression are problematic with autopsy samples alone. iPSC-derived neurons grown in culture allow for the detection of specific molecular and temporal signatures from mutation-carrying patients, thereby improving our understanding of the pathogenesis of tauopathies.

One limitation of using iPSCs is the inability to carry out studies under genetically defined conditions, leading to increased experimental variability. To overcome these obstacles, we used a zinc-finger-nuclease (ZFN)-mediated gene-editing technique (An et al., 2012; Corti et al., 2012; Li et al., 2012; Soldner et al., 2011) to generate isogenic human iPSC lines from an individual carrying a TAU-A152T mutation. We demonstrate that isogenic TAU-iPSCs are not only useful for modeling tauopathies, but also for identifying unknown molecular mechanisms underlying the disease-causing mutations.

## Results

### Generation of iPSCs from an Individual with a TAU-A152T Mutation

**Figure 1:**
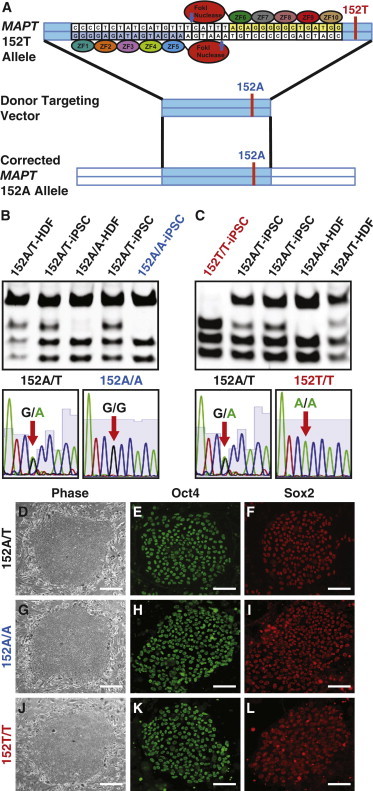
Generation of Isogenic Human TAU-A152T-iPSC Lines with ZFN-Mediated Gene-Editing Technology (A) Schematic of ZFN-mediated correction of the TAU-A152T mutation. (B) Screening of iPSCs with genetic correction of the TAU-A152T mutation by HphI restriction digestion of a TAU PCR product from genomic DNA (gDNA) of different iPSC clones. Sequencing of the gDNA from the isogenic TAU-152A/A-iPSCs confirmed correction of the nucleotide from A to G at the original mutation site (red arrows). For more information, see Figure S1. (C) Screening of iPSCs with engineered homozygous TAU-A152T mutation by HphI restriction digestion of a TAU PCR product from gDNA of different iPSC clones. Sequencing of the gDNA from the isogenic TAU-152T/T-iPSCs confirmed the change in nucleotide from G to A to generate a homozygous mutation (red arrows). For more information, see Figure S1. (D–L) Isogenic TAU-A152T-iPSCs demonstrated similar ES cell morphology (D, G, and J) and stained positive for pluripotency markers Oct4 (E, H, and K) and Sox2 (F, I, and L). Scale bars represent 50 μm. See also Figure S1.

**Figure 2:**
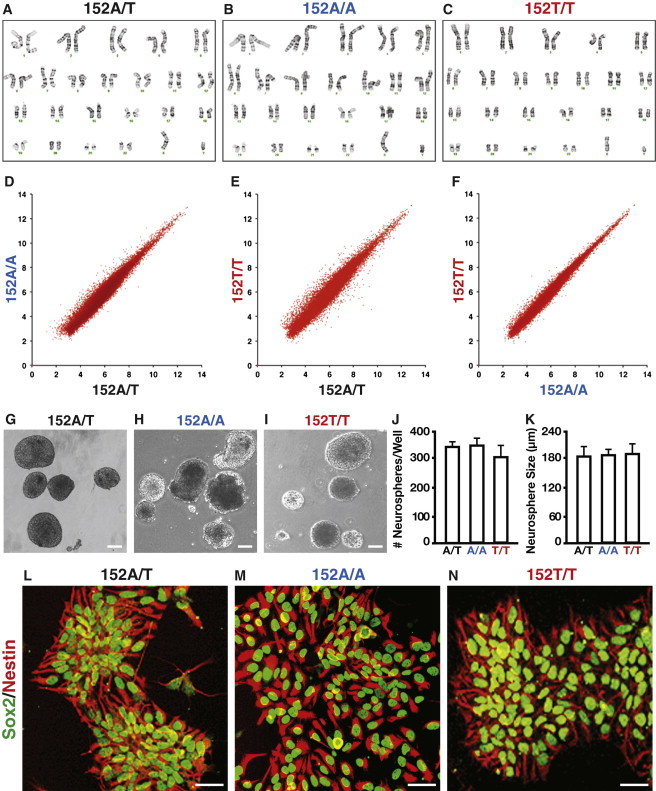
Characterization of the Isogenic TAU-A152T-iPSC Lines (A–C) Karyotype and G-band analysis confirmed a normal 46, XY karyotype in all three isogenic TAU-A152T-iPSC lines. (D–F) Global gene expression profiling demonstrated very few variations among all three isogenic TAU-A152T-iPSC lines. (G–K) All three isogenic TAU-A152T-iPSC lines generated neurospheres at similar levels and of comparable sizes. (L–N) Stable NSCs expressing the neural precursor markers Sox2 (green) and Nestin (red) could be generated equally well from all three isogenic TAU-A152T-iPSC lines. Values are mean ± SD. Scale bars represent 100 μm (G–I) and 50 μm (L–N).

We generated iPSCs from an individual carrying a heterozygous TAU-A152T mutation (Coppola et al., 2012; Kara et al., 2012; Kovacs et al., 2011) using a protocol published previously (Takahashi et al., 2007; Takahashi and Yamanaka, 2006). The TAU-152A/T-iPSCs had characteristics similar to embryonic stem (ES) cells, including their ES cell-like morphology (Figure 1D) and positive staining for ES cell markers (Figures 1E and 1F; Figures S1B–S1E available online). DNA sequencing confirmed a heterozygous TAU-A152T mutation (Figure 1B), and chromosomal analysis revealed a normal karyotype (Figure 2A). The TAU-152A/T-iPSCs formed teratomas in immunodeficient mice (Figures S1N–S1P), confirming their pluripotency.

### Generation of Isogenic TAU-A152T-iPSC Lines

We used ZFN-mediated gene-editing technology to generate isogenic TAU-A152T-iPSC lines (Hockemeyer et al., 2009; Miller et al., 2007; Soldner et al., 2011). The ZFN was designed to target ∼30 base pairs upstream of the A152T mutation on the *MAPT* gene (Figure 1A). We also used a 1,500 bp linear donor DNA fragment carrying the wild-type nucleotide sequence at position 152 as a repair template for homologous recombination (Figure 1A). Alternatively, a donor DNA fragment with a TAU-A152T mutation could be used to generate a homozygous TAU-A152T mutation. To obtain “scarless” genome editing, the donor DNA fragment contained no selection marker or excisable sequence. After nucleofection of the TAU-152-ZFN and the donor DNA fragment into 2 × 10^6^ TAU-152A/T-iPSCs (Fong et al., 2011), the cells were allowed to recover for 2 days and then were plated in a single-cell format in 96-well plates for 7–14 days.

Inspection of the DNA sequence of the TAU PCR product revealed two restriction cleavage sites for HphI on the wild-type allele and three sites on the mutant allele (Figure S1A). After screening 107 clones, a corrected clone was identified (Figure 1B, 152A/A-iPSC). From screening an additional 86 clones, we also found a clone containing a homozygous version of the TAU-A152T mutation, where the mutation was present on both alleles (Figure 1C, 152T/T-iPSC). DNA sequencing confirmed the correction or homozygous mutation of TAU-A152T in the isogenic iPSC lines (Figures 1B and 1C). The overall efficiency of TAU-ZFN-mediated gene editing was 1%−1.5%.

### Isogenic TAU-A152T-iPSC Lines Have Similar Genetic and Neural Stem Cell Differentiation Properties

Both isogenic TAU-152A/A-iPSC and TAU-152T/T-iPSC lines exhibited similar ES cell morphology to the parental TAU-152A/T-iPSC line and stained positive for pluripotency markers as seen in the parental iPSC line (Figures 1D–1L and S1B–S1M). Furthermore, the newly generated isogenic iPSC lines had a similar ability as the parental iPSC line to form teratomas in immunodeficient mice (Figures S1N–S1V), suggesting similar pluripotency among all three iPSC lines.

Karyotyping analysis showed that all three isogenic iPSC lines had a normal 46, XY karyotype (Figures 2A–2C). Global gene expression profiling of all three lines showed very few differences in gene expression patterns (Figures 2D–2F). Importantly, all three isogenic iPSC lines had similar abilities to generate neurospheres (Figures 2G–2K) and neural stem cells positive for Sox2 and Nestin (Figures 2L–2N).

### Genetic Correction of TAU-A152T Mutation Abolishes and Homozygous TAU-A152T Mutation Intensifies Tauopathy Phenotypes

**Figure 3:**
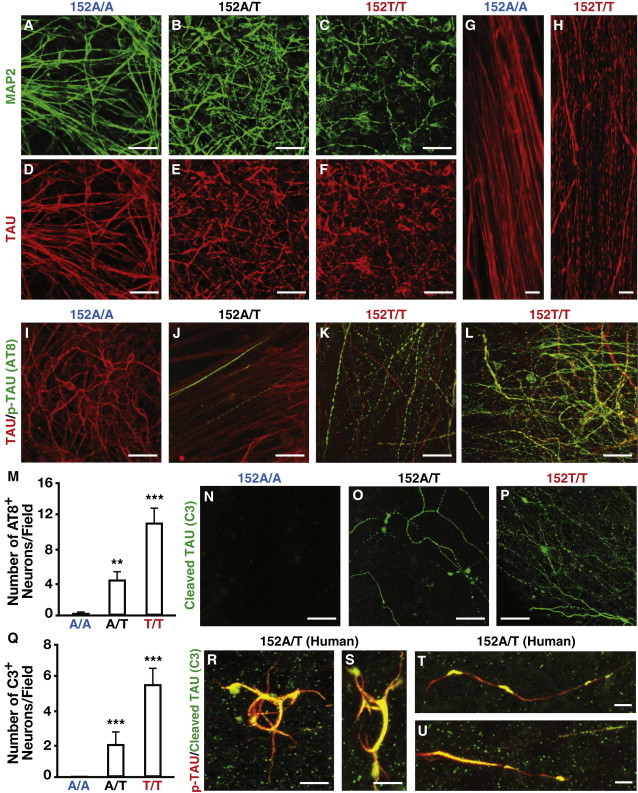
Genetic Correction of TAU-A152T Mutation Abolishes and Homozygous TAU-A152T Mutation Intensifies Tauopathy Phenotypes in iPSC-Derived Neurons as Determined by Immunocytochemistry (A–F) Mild neurodegeneration and accumulation of punctate TAU were found in neurons derived from TAU-152A/T-iPSCs (B and E), which were abolished in the mutation-corrected TAU-152A/A-iPSC-derived neurons (A and D) and intensified in homozygous TAU-152T/T-iPSC-derived neurons (C and F). (G and H) High magnification of TAU-152T/T-iPSC-derived neurons showed severe axonal degeneration with the accumulation of punctate TAU (H), which was completely rescued by genetic correction of the mutation in TAU-152A/A-iPSC-derived neurons (G). (I–L) AT8-positive phosphorylated TAU (p-TAU) was found in the axons of some neurons from TAU-152A/T-iPSCs (J, green). This phenotype was intensified in the axons and cell soma of neurons derived from TAU-152T/T-iPSCs (K and L, green). Correction of the mutation eliminates the phenotype as evidenced by little to no p-TAU in neurons derived from TAU-152A/A-iPSCs (I). All neurons were also costained for total TAU (red). (M) Quantification of AT8-positive neurons derived from the isogenic TAU-A152T-iPSC lines. (N–P) Caspase-cleaved TAU (C3) was found in the axons of neurons derived from TAU-152A/T-iPSCs (O), which was intensified in neurons derived from TAU-152T/T-iPSCs (P), but not in neurons derived from TAU-152A/A-iPSCs (N). (Q) Quantification of C3-positive neurons derived from the isogenic TAU-A152T-iPSC lines. (R–U) Caspase-cleaved TAU (C3, green) and p-TAU (red) were also found in the axons and cell soma of neurons from a patient with PSP carrying the TAU-A152T mutation. Values are mean ± SD. Scale bars represent 30 μm (A–F, I–L, and N–P), 5 μm (G and H), and 10 μm (R–U). See also Figure S2.

Upon neuronal differentiation of the parental TAU-152A/T-iPSCs (Chambers et al., 2009; Hu and Zhang, 2009; Ring et al., 2012), immunocytochemical analysis of TAU revealed unhealthy looking neurons with punctate TAU staining in neuronal processes (Figure 3E). Their neurites were short and tapering and appeared to bulge and constrict with odd bends and breaks (Figure 3E). MAP2 immunostaining confirmed mild degeneration of neuronal processes (Figure 3B). Genetic correction of the mutation in the isogenic TAU-152A/A-iPSCs abolished the neurodegenerative phenotype, leading to the generation of healthy looking neurons with smooth TAU and MAP2 staining (Figures 3A and 3D). In sharp contrast, the isogenic homozygous TAU-152T/T-iPSCs generated neurons with severe degeneration and a much lower survival of neurons, as indicated by MAP2 staining (Figure 3C), and much more punctate TAU staining in neuronal processes (Figure 3F). Quantitatively, there was a gene-dose-dependent effect of the TAU-A152T mutation on neurodegeneration as evidenced by abnormal TAU staining (Figure S2A). High-magnification images revealed severe axonal degeneration and fragmentation of neurons derived from TAU-152T/T-iPSCs (Figure 3H), which was completely rescued by correcting the mutation in the isogenic TAU-152A/A-iPSCs (Figure 3G). Neurodegeneration was also observed in a subclone of TAU-152T/T-iPSCs (152T/T-2), as evidenced by abnormal TAU staining (Figure S2B). Low-density neuronal culture and double immunostaining for TAU and MAP2 revealed mislocalization of TAU-A152T to the somatodendritic domains in TAU-152T/T-iPSC-derived neurons (Figures S2E and S2F).

**Figure 4:**
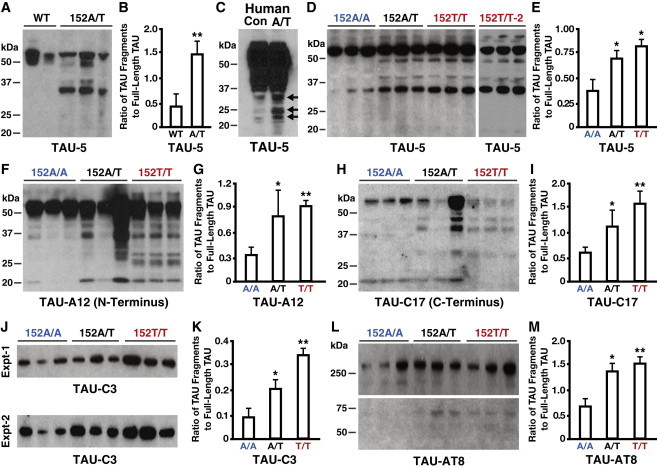
Genetic Correction of TAU-A152T Mutation Abolishes and Homozygous TAU-A152T Mutation Intensifies Tauopathy Phenotypes in iPSC-Derived Neurons as Determined by Western Blotting (A and B) Western blotting with the TAU-5 antibody revealed more TAU fragmentation in neurons derived from the parental TAU-152A/T-iPSCs than in neurons derived from control iPSCs without the mutation (A). Quantification of TAU bands showed a significantly greater amount of total fragments of TAU in TAU-152A/T-iPSC-derived neurons than in the control iPSC-derived neurons (B). (C) Western blotting with the TAU-5 antibody also revealed increased fragmentation of the TAU protein (arrows) in the brain lysate of a patient with PSP carrying the TAU-A152T mutation. (D–I) Western blotting with an antibody against the central part (D and E, TAU-5), the N terminus (F and G, TAU-A12), or the C terminus (H and I, TAU-C17) of the TAU protein confirmed the increases in TAU fragmentation in neurons derived from TAU-152A/T-iPSCs and TAU-152T/T-iPSCs. The numbers and amounts of TAU fragmentations were dramatically less upon genetic correction of the mutation. (J and K) A mutant gene-dose-dependent increase of caspase-cleaved TAU (TAU-C3) was observed in neurons derived from the three isogenic TAU-152A/T-iPSC lines. (L and M) Higher levels of AT8-positive p-TAU at two molecular masses (>250 and ∼74 kDa) were found in neurons derived from TAU-152A/T-iPSCs and TAU-152T/T-iPSCs than in neurons derived from TAU-152A/A-iPSCs. All TAU band quantifications were normalized to total TAU. Values are mean ± SD. ^∗^p ≤ 0.05, ^∗∗^p ≤ 0.01.

AT8-positive phosphorylated TAU (p-TAU) was found in some neurons derived from TAU-152A/T-iPSCs, with a predominant and punctate location in axons (Figure 3J). Again, genetic correction of the mutation abolished p-TAU accumulation (Figure 3I), whereas the homozygous mutation exacerbated p-TAU accumulation in both axons and cell soma (Figures 3K and 3L). The mutation-related increase in p-TAU was also observed in a subclone of TAU-152T/T-iPSC-derived neurons (Figure S2C). Strikingly, the numbers of p-TAU-positive neurons increased in a TAU-A152T gene-dose-dependent manner (Figure 3M). Accumulation of p-TAU was restricted to neurons as there was no p-TAU in GFAP-positive astrocytes (Figure S2G). Western blotting analysis confirmed the presence of high levels of p-TAU in neurons derived from TAU-152A/T-iPSCs or TAU-152T/T-iPSCs, which was dramatically decreased in neurons derived from TAU-152A/A-iPSCs (Figures 4L and 4M).

### Genetic Correction of TAU-A152T Mutation Eliminates and Homozygous TAU-A152T Mutation Intensifies the Generation of Pathological TAU Fragments in Neurons

We observed a greater degree of TAU fragmentation, as determined by western blotting using the antibody TAU-5, in neurons derived from TAU-152A/T-iPSCs than those derived from unrelated control iPSCs without the mutation (Figures 4A and 4B). More TAU fragmentation was also observed in brain samples from a patient with PSP carrying the TAU-A152T mutation than in those from a control subject without the variant (Figure 4C, arrows). Importantly, the mutation-related increase in TAU fragmentation in human iPSC-derived neurons was confirmed by western blotting with three different TAU antibodies (TAU-5, TAU-A12, and TAU-C17 that recognize the central core, N terminus, and C terminus of the protein, respectively) (Carmel et al., 1996; Porzig et al., 2007) and was decreased dramatically upon genetic correction of the mutation (Figures 4D–4I). The mutation-related increase in TAU fragmentation was confirmed in neurons derived from a subclone of TAU-152T/T-iPSCs (Figure 4D, 152T/T-2).

Most strikingly, there was a mutant gene-dose-dependent increase in caspase-cleaved TAU fragments, as determined by the caspase-cleaved TAU-specific antibody C3 (Gamblin et al., 2003; Guillozet-Bongaarts et al., 2005), in neurons derived from the isogenic TAU-A152T-iPSCs (Figures 4J and 4K). In line with this observation, immunostaining with the TAU-C3 antibody revealed the accumulation of caspase-cleaved TAU in axons of neurons derived from TAU-152A/T-iPSCs and TAU-152T/T-iPSCs (Figures 3O and 3P) but not in those of neurons derived from TAU-152A/A-iPSCs (Figure 3N). The mutation-related accumulation of caspase-cleaved TAU was also observed in neurons derived from a subclone of TAU-152T/T-iPSCs (Figure S2D). The numbers of caspase-cleaved TAU-positive neurons increased in a mutant gene-dose-dependent manner (Figure 3Q). We observed no caspase-cleaved TAU in GFAP-positive astrocytes (Figure S2H). Likewise, caspase-cleaved TAU also accumulated in neuronal soma and axons in the patient with PSP carrying the TAU-A152T mutation (Figures 3R–3U).

### Genetic Correction of TAU-A152T Eliminates the Detrimental Effects of the Mutation on Different Subtypes of Neurons

We then determined the effects of the TAU-A152T mutation on different subtypes of neurons, including tyrosine-hydroxylase (TH)-positive dopaminergic neurons, T-box brain 1 (TBR1)-positive glutamatergic excitatory neurons (Hevner et al., 2001), and gamma-aminobutyric-acid (GABA)-positive inhibitory neurons. Strikingly, very low percentages of dopaminergic neurons were found in neuronal cultures from TAU-152A/T-iPSCs and TAU-152T/T-iPSCs (Figures S2J–S2L). Genetic correction of the mutation increased the percentages of dopaminergic neurons by 4- to 8-fold (Figures S2I–S2L), suggesting that dopaminergic neurons are especially vulnerable to TAU-A152T-induced neurotoxicity. Interestingly, the percentages of glutamatergic and GABAergic neurons were not significantly altered by the mutation (Figures S2M–S2T). However, many glutamatergic (Figure S2V) and GABAergic (Figure S2X) neurons had abnormal morphologies, including neurite fragmentation/degeneration, which were also eliminated by genetic correction of the mutation (Figures S2U and S2W).

## Discussion

By combining the iPSC and ZFN-mediated gene-editing techniques, we generated “scarless” isogenic human iPSC lines carrying wild-type TAU or a heterozygous or homozygous TAU-A152T mutation. The use of genetically matched isogenic iPSC lines, with three gene doses of the mutation (zero, one, and two copies) on an identical genetic background, eliminates potential subject-to-subject and line-to-line variations of iPSCs and helps draw a clear conclusion regarding mutation-specific phenotypes. The isogenic TAU-iPSC lines generated in this study will be invaluable for further mechanistic studies of tauopathies and for related drug-screening efforts.

Before the TAU-A152T mutation was identified in humans, it was widely accepted that mutations of TAU cause FTD and PSP, but not AD (Huang and Mucke, 2012). The TAU-A152T mutation is the first to show the association of a TAU mutation with increased risks for FTD, PSP, and AD (Coppola et al., 2012). Thus, the tauopathy model of human iPSCs, and the related molecular mechanisms identified in this study, should be applicable to all three tauopathy-related diseases for further mechanistic studies and drug screening.

Although modeling neurodegenerative diseases with iPSCs has been reported (HD iPSC Consortium, 2012; Israel et al., 2012; Kondo et al., 2013; Park et al., 2008; Yamanaka, 2009), they typically recapitulate phenotypes reported in cell cultures or animal studies. Our study, however, identifies an unknown molecular mechanism underlying a disease-causing TAU mutation using isogenic iPSCs. A previous study reported that the TAU-A152T mutation increased soluble TAU oligomers and decreased microtubule-binding affinity of TAU in cell cultures and test tubes (Coppola et al., 2012). We demonstrate in iPSC-derived human neurons that this mutation predisposes TAU to proteolysis by caspase and other proteases, leading to tauopathy, axonal degeneration, and other related pathologies. We further demonstrate that correction of the mutation eliminates TAU proteolysis, indicating the specific effect of the mutation on TAU proteolysis. Our work therefore directly demonstrates that iPSCs, especially isogenic iPSCs, are a powerful tool not only for disease modeling but also for studies of disease mechanisms. Interestingly, dopaminergic neurons are especially vulnerable to TAU-A152T-induced neurotoxicity, which is in line with the observation that tauopathy in PSP is associated with excessive dopaminergic neuron loss (Murphy et al., 2008). The underlying mechanism remains to be determined.

## Experimental Procedures

### Reprogramming Human Dermal Fibroblasts into iPSCs

Fibroblasts were obtained from an individual carrying the TAU-A152T mutation in the *MAPT* gene. iPSCs were generated from early passages of fibroblasts by a retroviral reprogramming strategy with four factors (Oct4, Sox2, Klf4, and c-Myc) (Takahashi et al., 2007; Takahashi and Yamanaka, 2006). The animal procedure for testing teratoma formation was approved by the Gladstone Institutes and the University of California, San Francisco.

### Preparation of ZFNs and Generation of Isogenic iPSC Lines

A specific pair of five-finger ZFNs engineered to target a region ∼30 bp upstream of the A152T mutation site was prepared by Sigma. The ZFNs bound (uppercase) and cut (lowercase) the following sequence on the *MAPT* gene: CCCCTCTATCATGTTtcatttACAGGGGGCTGATGG. Isogenic TAU-A152T-iPSC lines were generated using this ZFN pair and a donor construct (An et al., 2012; Corti et al., 2012; Li et al., 2012; Soldner et al., 2011).

### Neuronal Differentiation of iPSCs

iPSCs were differentiated into neurons following a modified version of published protocols (Chambers et al., 2009; Hu and Zhang, 2009; Ring et al., 2012). Tauopathies were characterized by western blotting and immunocytochemistry with different TAU antibodies.

### Human Neuropathology

Tauopathies were characterized by western blotting and immunocytochemistry with different TAU antibodies on postmortem brain tissues from a 56-year-old woman with clinical and pathological PSP who carried the *MAPT* A152T variant and a 76-year-old man who died of prostate cancer without cognitive complaints.

### Statistical Analyses

Values are expressed as mean ± SD. Differences between means were assessed by t test or analysis of variance (ANOVA). p < 0.05 was considered statistically significant.

Detailed methods, including isogenic iPSC generation and characterization, neuronal differentiation, immunostaining, western blotting, and tauopathy analyses can be found in the Supplemental Information.

## Supplemental Information

Document S1. Supplemental Experimental Procedures, Figures S1 and S2, and Table S1
