# Supplementary material for: Genetic Correction of Tauopathy Phenotypes in Neurons Derived from Human Induced Pluripotent Stem Cells
Source: Stem Cell Reports. 2013 Aug 29;1(3):226–34. doi: 10.1016/j.stemcr.2013.08.001 (PMC3849235; doi:10.1016/j.stemcr.2013.08.001)
Supplement: Document S1. Supplemental Experimental Procedures, Figures S1 and S2, and Table S1 [file mmc1.pdf]

Stem Cell Reports, Volume *1*

Supplemental Information

Genetic Correction of Tauopathy Phenotypes in Neurons Derived from Human Induced Pluripotent Stem Cells

Helen Fong, Chengzhong Wang, Johanna Knoferle, David Walker, Maureen E. Balestra, Leslie M. Tong, Laura Leung, Karen L. Ring, William W. Seeley, Anna Karydas, Mihir A. Kshirsagar, Adam L. Boxer, Kenneth S. Kosik, Bruce L. Miller, and Yadong Huang

Inventory of Supplemental Information

Figure S1 related to Figure 1

Figure S2 related to Figure 3

Table S1

Supplemental Experimental Procedures

Supplemental References

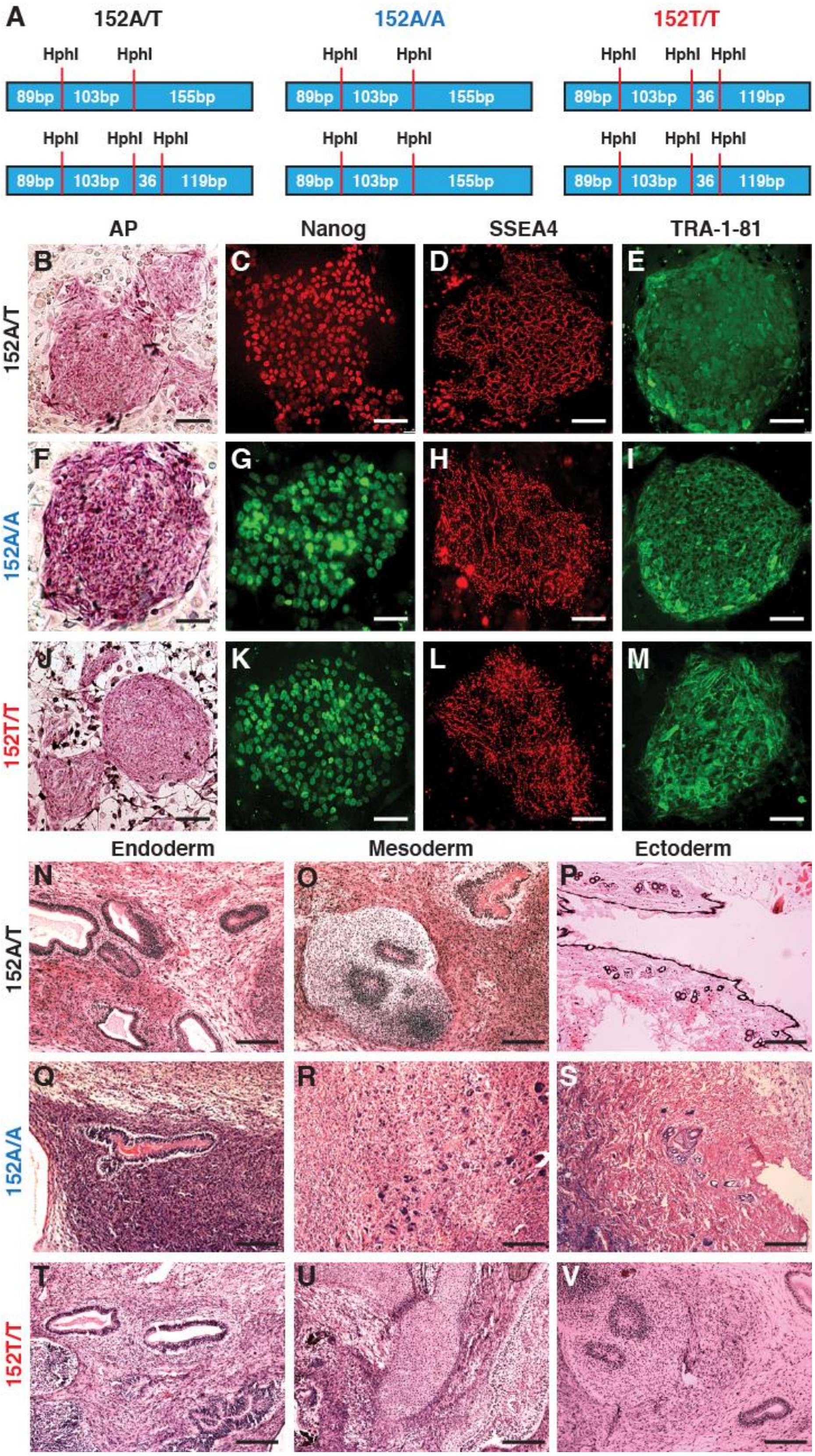
A
152A/T
152A/A
152T/T
HphI
89bp
103bp
155bp
36
119bp
AP
Nanog
SSEA4
TRA-1-81
B
C
D
E
F
G
H
I
J
K
L
M
Endoderm
Mesoderm
Ectoderm
N
O
P
Q
R
S
T
U
V

Figure S1, Related to Figure 1. Generation of the isogenic TAU-A152T-iPSC lines

(A) Schematic of the ZFN-targeted region of the *MAPT* allele showed two HphI restriction cleavage sites on the wild-type allele and three on the mutant allele. Upon correction of the TAU-A152T mutation, one restriction site on the mutant allele was eliminated. When a homozygous mutation was generated, an additional restriction site was added to the wild-type allele.

(B–M) All three isogenic TAU-A152T-iPSC lines expressed ES cell markers, including alkaline phosphatase (AP, B, F, and J), the nuclear marker Nanog (C, G, and K), and the surface markers SSEA4 (D, H, and L) and TRA-1-81 (E, I, and M).

(N–V) H&E staining of teratoma sections from the isogenic TAU-152A/T-iPSCs (N–P), TAU-152A/A-iPSCs (Q–S), and TAU-152T/T-iPSCs (T–V) showed the development of endoderm (N, Q, and T), mesoderm (O, R, and U), and ectoderm (P, S, and V).

Scale bars represent 50 μm in (B)–(M) and 200 μm in (N)–(V).

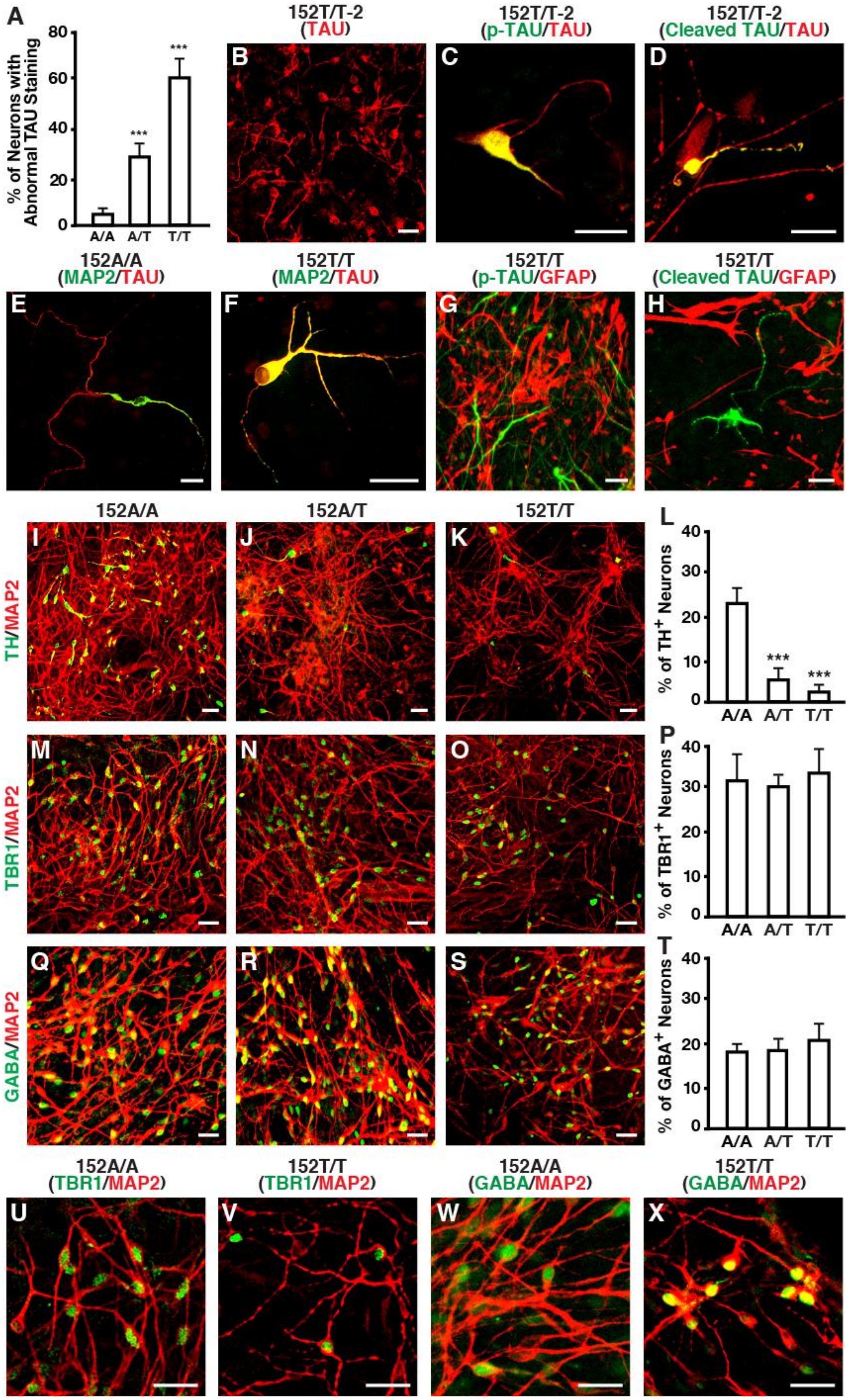
A
% of Neurons with Abnormal TAU Staining
80
60
40
20
0
A/A
A/T
T/T
***
***
152T/T-2
(TAU)
B
152T/T-2
(p-TAU/TAU)
C
152T/T-2
(Cleaved TAU/TAU)
D
152A/A
(MAP2/TAU)
E
152T/T
(MAP2/TAU)
F
152T/T
(p-TAU/GFAP)
G
152T/T
(Cleaved TAU/GFAP)
H
152A/A
152A/T
152T/T
TH/MAP2
I
J
K
L
% of TH$^+$ Neurons
40
30
20
10
0
A/A
A/T
T/T
TBR1/MAP2
M
N
O
P
% of TBR1$^+$ Neurons
GABA/MAP2
Q
R
S
T
% of GABA$^+$ Neurons
152A/A
(TBR1/MAP2)
U
152T/T
(TBR1/MAP2)
V
152A/A
(GABA/MAP2)
W
152T/T
(GABA/MAP2)
X

Figure S2, Related to Figure 3. Genetic correction of the TAU-A152T mutation abolishes and homozygous TAU-A152T mutation intensifies tauopathy phenotypes in iPSC-derived neurons

(A) Quantification of neurons with abnormal TAU staining shows a gene-dose dependent effect of the TAU-A152T mutation.

(B) Neurodegeneration and accumulation of punctate TAU were found in neurons derived from a subclone of TAU-152T/T-iPSCs (152T/T-2).

(C) AT8-positive phosphorylated TAU (p-TAU, green) was found in neurons derived from a subclone of TAU-152T/T-iPSCs (152T/T-2). Neurons were also co-stained for total TAU (red).

(D) Caspase-cleaved TAU (C3, green) was found in neurons derived from a subclone of TAU-152T/T-iPSCs (152T/T-2). Neurons were also co-stained for total TAU (red).

(E, F) Somatodendritic mislocalization of TAU in neurons derived from TAU-152T/T-iPSCs (F), which was abolished in neurons derived from TAU-152A/A-iPSCs (E).

(G, H) p-TAU (G) and caspase-cleaved TAU (H) (both green) were not found in TAU-152T/T-iPSC-derived astrocytes (GFAP, red).

(I–L) Immunostaining and quantification of TH-positive dopaminergic neurons (green) derived from three isogenic iPSC lines show lower percentages of TH-positive neurons derived from TAU-152A/T-iPSCs and TAU-152T/T-iPSCs. Neurons were also co-stained for MAP2 (red).

(M–P) Immunostaining and quantification of TBR1-positive glutamatergic excitatory neurons (green) derived from three isogenic iPSC lines show equivalent percentages of TBR1-positive neurons. Neurons were also co-stained for MAP2 (red).

(Q–T) Immunostaining and quantification of GABA-positive inhibitory neurons (green) derived from three isogenic iPSC lines show equivalent percentages of GABA-positive neurons. Neurons were also co-stained for MAP2 (red).

(U, V) High-magnification images of TBR1-positive glutamatergic excitatory neurons (green) derived from TAU-152A/A-iPSCs (U) and TAU-152T/T-iPSCs (V) show abnormal morphology of neurons carrying the TAU-152T/T mutation. Neurons were also co-stained for MAP2 (red).

(W, X) High-magnification images of GABA-positive inhibitory neurons (green) derived from TAU-152A/A-iPSCs (W) and TAU-152T/T-iPSCs (X) show abnormal morphology of neurons carrying the TAU-152T/T mutation. Neurons were also co-stained for MAP2 (red).

Values are mean ± SD. Scale bars represent 20 μm.

Table S1. Primary antibodies used for immunostaining and western blot analyses.

**Name**	**Dilution**	**Supplier**
OCT4	1:40	R & D Systems
SOX2	1:40	R & D Systems
Nanog	1:1000	Abcam
SSEA4	1:1000	Developmental Studies Hybridoma Bank
TRA-1-81	1:100	Santa Cruz Biotechnology, Inc.
Nestin	1:200	Millipore
MAP2 (from mouse)	1:200	Millipore
MAP2 (from rabbit)	1:1000	Millipore
TAU (total)	1:1000	Sigma
TAU-5	1:2000	BD Pharmingen
TAU-AT8	1:500 (ICC)<br>1:1000 (WB)	Thermo
Biotinylated TAU-AT8	1:500	Millipore
TAU-C3	1:200 (ICC)<br>1:1500 (WB)	Millipore
TAU-A12	1:100	Santa Cruz Biotechnology, Inc.
TAU-C17	1:200	Santa Cruz Biotechoology, Inc.
GABA	1:1000	Sigma
TH	1:1000	Millipore
TBR1	1:250	Abcam
GFAP	1:1000	Dako

SUPPLEMENTAL EXPERIMENTAL PROCEDURES

Reprogramming human dermal fibroblasts into iPSCs

Fibroblasts were obtained from an individual carrying the TAU-A152T mutation in the *MAPT* gene. The donor is heterozygous for a G to A transition at nucleotide 102,063 (NCBI Reference Sequence: NG_007398.1) in exon 7 of *MAPT* that converts alanine 152 (GCC) to threonine (ACC) (Coppola et al., 2012; Kara et al., 2012; Kovacs et al., 2011). iPSCs were generated from early passages of fibroblasts by a retroviral reprogramming strategy, as reported (Takahashi et al., 2007; Takahashi and Yamanaka, 2006). Briefly, fibroblasts were infected with pseudotyped retroviral vectors expressing the four transcription factors Oct4, Sox2, Klf4, and c-Myc. After infection, fibroblasts were seeded onto irradiated SNL feeder cells for three to four weeks under human embryonic stem cell culture conditions. iPSC colonies were then identified and mechanically isolated.

Teratoma and karyotype analyses of iPSCs

For teratoma formation, $1x10^6$ cells of each iPSC line (TAU-152A/T, TAU-152A/A, and TAU-152T/T) were briefly dissociated with Accutase (Millipore), spun down at 1000 rpm, and resuspended in 100 μl of PBS. NOD-SCID mice were anesthetized by standard procedure. The cell suspensions were injected into the right and left thigh muscles ($1x10^6$ cells per site) of three mice. After injections, mice were monitored for recovery. Tumors were apparent by about 4 weeks. After collection, the tumors were fixed, sectioned, and stained for H&E to identify teratomas. For karyotype analysis, each iPSC line was karyotyped by Cell Line Genetics and the UCSF Cytogenetics Lab.

Preparation of ZFN targeting a position close to A152T in human *MAPT* gene

ZFN-mediated gene editing was achieved by introducing a double-strand DNA break in a target region and by providing a donor construct containing the desired and modified sequence. A specific pair of five-finger ZFNs engineered to target a region ~30 bp upstream of the A152T mutation site was prepared by Sigma. The ZFNs bound (uppercase) and cut (lowercase) the following sequence on the *MAPT* gene: CCCCTCTATCATGTTtcatttACAGGGGGCTGATGG. The TAU-ZFNs were first tested in human K562 cells to assure effectiveness in generating a double-strand DNA break at the correct site in the human *MAPT* gene. We generated a 1500-bp

donor construct containing the desired and modified nucleotide sequence spanning both sides of the mutation site.

Nucleofection of TAU-ZFN mRNAs and the donor DNA

All nucleofections were performed using the Nucleofector II (Lonza) with Nucleofector setting A-23 and the Human Stem Cell Nucleofector Kit I following a published protocol (Fong et al., 2011; Hohenstein et al., 2008). iPSCs were dissociated with Accutase (Millipore) and 2×10^6 cells were used for each nucleofection. 5 μg of the TAU-ZFN mRNA pair and 5 μg of donor DNA were mixed with the cells in the nucleofection solution. The mixture was transferred to a cuvette, nucleofected, and quickly transferred into 500 μl of prewarmed (37°C) hES medium. Nucleofected cells were incubated at 37°C for 5 minutes and plated at 1×10^6 cells per well in a SNL-coated six-well plate containing hES medium supplemented with ROCK inhibitor (ROCKi) (Tocris). To maximize viability and efficiency, the entire procedure was completed within 20 minutes. To determine transfection efficiency, 5 μg of pMAX-GFP vector was nucleofected into a parallel set of cells to monitor GFP expression.

iPSC culture and cloning

iPSCs were maintained on irradiated SNL feeder cells in hES medium consisting of DMEM/F12 with 20% Knockout Serum Replacement supplemented with 2 mM glutamine, 0.1 mM nonessential amino acids, 4 ng/ml recombinant human bFGF (all Life Technologies), and 0.1 mM 2-mercaptoethanol (2-ME) (Takahashi et al., 2007). For passaging, iPSC colonies were detached by brief treatment with Accutase and scraped. Cells were routinely passaged at a 1:2–1:4 split ratio. For clonal isolation of modified iPSCs after nucleofection, cells were completely dissociated into single cells with Accutase and plated at 1 cell/well in a 96-well plate coated with irradiated SNL feeders. Clonal iPSCs were maintained in hES medium supplemented with 10 μM of ROCKi for the first week of culture. Single colonies appearing in the 96-well plate were passaged into 48-well plates followed by expansion into 24-well plates. Cells were then harvested for genomic DNA (gDNA) isolation.

Screening of genetically modified iPSC clones using a HphI assay

gDNA was isolated from iPSC clones with GenElute Mammalian Genomic DNA Miniprep Kit (Sigma), following manufacturer's instructions. 250 ng of gDNA was PCR-amplified using

Phusion High Fidelity DNA Polymerase (Thermo) with 0.5 μM forward and reverse primers flanking the ZFN binding site and A152T mutation on the *MAPT* gene to yield a 347-bp fragment. Forward and reverse primer sequences are as follows: Forward 5'-TCAGGGAACTTGGAGTTTGG-3' and Reverse 5'-TTCTTACCAGAGCTGGGTGG-3'. Genomic DNA from individual colonies was screened using the HphI restriction enzyme to assess changes specifically at the mutation site. Inspection of the DNA sequence of the TAU PCR product revealed two restriction cleavage sites for HphI on the wild-type allele and three sites on the mutant allele (Fig. S1A). An additional cleavage site was present as a result of the TAU-A152T mutation. Upon correction of the mutation, the additional HphI site would be eliminated (Fig. S1A). In contrast, the occurrence of a homozygous TAU-A152T mutation would generate an additional cleavage site on the wild-type allele (Fig. S1A). Thus, the loss and gain of an HphI restriction site allowed for the efficient identification of genetically modified clones with the mutation corrected or a homozygous mutation engineered (Fig. S1A). For digestion with HphI restriction enzyme, PCR products were incubated in NEBuffer #4 for 4 hours at 37°C and inactivated by incubation for 20 minutes at 65°C. Digested PCR products were separated on a 4−15% TBE gel.

Neuronal differentiation of iPSCs

iPSCs were differentiated into neurons by following a modified version of published protocols (Chambers, 2009; Hu, 2009; Ring, 2011). iPSCs were grown in suspension as embryoid bodies (EBs) in hES medium without bFGF after treatment with Collagenase IV (Stem Cell Technologies). Medium was replaced every day for 5 days. On day 5, EBs were grown in Neural Medium (DMEM/F12, Neurobasal media, 0.5% N2 Supplement (Life Technologies), 1% B27 Supplement (Life Technologies), non-essential amino acids, 1 mg/mL heparin (Sigma), and 0.5% penicillin/streptomycin (Life Technologies) supplemented with 5 μM SB431542 (Stemgent) and 0.25 μM LDN-193189 (Stemgent). On day 7, spheres were transferred to Matrigel (BD Biosciences)-coated wells for attachment and grown in Neural Medium without SB and LDN. Attached neuroepithelial cells were fed every other day for 7 days. By day 15, neural rosette structures appeared and were mechanically lifted off the plate by quick expulsion of medium through a P1000 pipette. Lifted rosettes were then grown in suspension in Neural Medium with the addition of 5 ng/ml bFGF and 20 ng/ml EGF for an additional 2 weeks. For neuronal differentiation, rosettes were plated down on culture plates coated with 100 μg/mL of poly-l-

ornithine (Sigma) and 200 μg/mL of laminin (Sigma). Neurons were observed after several days and were differentiated further for an additional 30 days or longer after plating. Neuronal cultures were maintained in Neural Medium in the absence of bFGF and EGF.

Western blotting analysis

iPSC-derived neurons were collected for western blotting by washing twice with PBS and scraping with a cell scraper. Cells were spun down in 1.5 ml microcentrifuge tubes and homogenized in the presence of a high-detergent buffer (50 mM Tris, 150 mM sodium chloride, 2% Nonidet P-40, 1% sodium deoxycholate, 4% SDS, and supplemented with Complete protease inhibitor cocktail (Roche), phosphatase inhibitor cocktail 1 (P2850, Sigma), and phosphatase inhibitor cocktail 2 (P5726, Sigma)) three times for 15 seconds each on ice (Li et al., 2009). Lysates were spun down at maximum speed in a microcentrifuge for 10 minutes at 4°C to remove cellular debris. The supernatant was collected, and total protein was quantitated using the BCA protein assay kit (Pierce). Total protein (15 μg) was denatured at 70°C for 10 minutes in 1X NuPAGE Reducing Agent and 1X NuPAGE LDS Sample Buffer, electrophoresed on a 4−20% Bis-Tris polyacrylamide gel (Life Technologies) and transferred to a nitrocellulose membrane (Biorad). Membranes were stained with Ponceau-S to determine equal sample loading and transfer. The membranes were then blocked in 5% milk in phosphate buffered saline containing 0.1% Tween-20 and probed with primary antibodies as listed in Supplemental Table 1 followed by a horseradish peroxidase–conjugated secondary antibody (Dako). All antibodies were diluted according to the manufacturer's instructions and visualized using an enhanced chemiluminescence kit (Pierce).

Immunocytochemical and imaging analyses

All cells were washed with 1X PBS (Life Technologies), fixed in 4% paraformaldehyde for 20 minutes at 4°C, and blocked in 10% normal serum with 0.5% Triton X-100 (Sigma) in PBS. Immunocytochemical analysis was performed using primary antibodies as listed in Supplemental Table 1 and corresponding AlexaFluor-conjugated secondary antibodies (Life Technologies). All antibodies were diluted and used according to manufacturer's instructions. Cell nuclei were counterstained with DAPI in some experiments. Images were collected using a Leica epifluorescent microscope, a Keyence BZ-9000E fluorescent microscope, or a BioRad confocal imaging system (Li et al., 2009; Ring et al., 2012).

Human neuropathology

Formalin-fixed and frozen post-mortem brain tissues were provided by the UCSF Neurodegenerative Disease Brain Bank from (1) a 56 year-old woman with clinical and pathological progressive supranuclear palsy (PSP) who carried the *MAPT* A152T variant and (2) a 76 year-old man who died of prostate cancer without cognitive complaints. Formalin-fixed brain tissues were placed in a 10–20–30% sucrose gradient for cryoprotection. Tissues (30 μm thickness) were cut with a sliding microtome into serial sections consisting of approximately 10 sections per series. Sections were co-stained with TAU-C3 and biotin-conjugated TAU-AT8 (Thermo) primary antibodies followed by corresponding AlexaFluor-conjugated secondary antibodies. All antibodies were diluted and used according to manufacturer's instructions. Frozen brain tissues were homogenized on ice 5X for 30 seconds in high detergent buffer supplemented with complete protease inhibitor cocktail (Roche), phosphatase inhibitor cocktail 1 (P2850), and phosphatase inhibitor cocktail 2 (P5726) using a Polytron homogenizer (Brinkman) (Li et al., 2009). The brain homogenates were then centrifuged at 38000 rpm for 1 hour, separated from the pellet, and stored at -80°C. Total protein was quantitated using the BCA protein assay kit and analyzed by western blot as previously described using the TAU-5 antibody (Li et al., 2009).

Statistical analyses

Values are expressed as mean ± SD. Differences between means were assessed by *t* test or analysis of variance (ANOVA). $P < 0.05$ was considered statistically significant.

Supplemental References

Chambers, S.M., Fasano, C.A., Papapetrou, E.P., Tomishima, M., Sadelain, M., and Studer, L. (2009). Highly efficient neural conversion of human ES and iPS cells by dual inhibiton of SMAD signaling. Nat Biotechnol *27*, 275–280.

Coppola, G., Chinnathambi, S., Lee, J.J., Dombroski, B.A., Baker, M.C., Soto-Ortolaza, A.I., Lee, S.E., Klein, E., Huang, A.Y., Sears, R., *et al.* (2012). Evidence for a role of the rare p.A152T variant in *MAPT* in increasing the risk for FTD-spectrum and Alzheimer's diseases. Hum Mol Genet *21*, 3500–3512.

Fong, H., Elliott, K.A., Lock, L.F., and Donovan, P.J. (2011). Nucleofection of human embryonic stem cells. Methods Mol Biol *767*, 333–341.

Hohenstein, K.A., Pyle, A.D., Chern, J.Y., Lock, L.F., and Donovan, P.J. (2008). Nucleofection mediates high-efficiency stable gene knockdown and trangene expression in human embryonic stem cells. Stem Cells *26*, 1436–1443.

Hu, B.Y., and Zhang, S.C. (2009). Differentiation of spinal motor neurons from pluripotent human stem cells. Nat Protoc *4*, 1295–1304.

Kara, E., Ling, H., Pittman, A.M., Shaw, K., Silva, R.d., Simone, R., Holton, J.L., Warren, J.D., Rohrer, J.D., Xiromerisiou, G., *et al.* (2012). The MAPT p.A152T variant is a risk factor associated with tauopathies with atypical clinical and neuropathological features. Neurobiol aging *33*, 2231.

Kovacs, G.G., Wöhrer, A., Ströbel, T., Botond, G., Attems, J., and Budka, H. (2011). Unclassifiable tauopathy associated with an A152T variation in MAPT exon 7. Clin Neuropathol *30*, 3–10.

Li, G., Bien-Ly, N., Andrews-Zwilling, Y., Xu, Q., Bernardo, A., Ring, K., Halabisky, B., Deng, C., Mahley, R.W., and Huang, Y. (2009). GABAergic interneuron dysfunction impairs hippocampal neurogenesis in adult apolipoprotein E4 knockin mice. Cell Stem Cell *5*, 634–645.

Ring, K.L., Tong, L.M., Balestra, M.E., Javier, R., Andrews-Zwilling, Y., Li, G., Walker, D., Zhang, W.R., Kreitzer, A.C., and Huang, Y. (2012). Direct reprogramming of mouse and human fibroblasts into multipotent neural stem cells with a single factor. Cell Stem Cell *11*, 100–109.

Takahashi, K., Tanabe, K., Ohnuki, M., Narita, M., Ichisaka, T., Tomoda, K., and Yamanaka, S. (2007). Induction of pluripotent stem cells from adult human fibroblasts by defined factors. Cell *131*, 861–872.

Takahashi, K., and Yamanaka, S. (2006). Induction of pluripotent stem cells from mouse embryonic and adult fibroblast cultures by defined factors. Cell *126*, 663–676.
